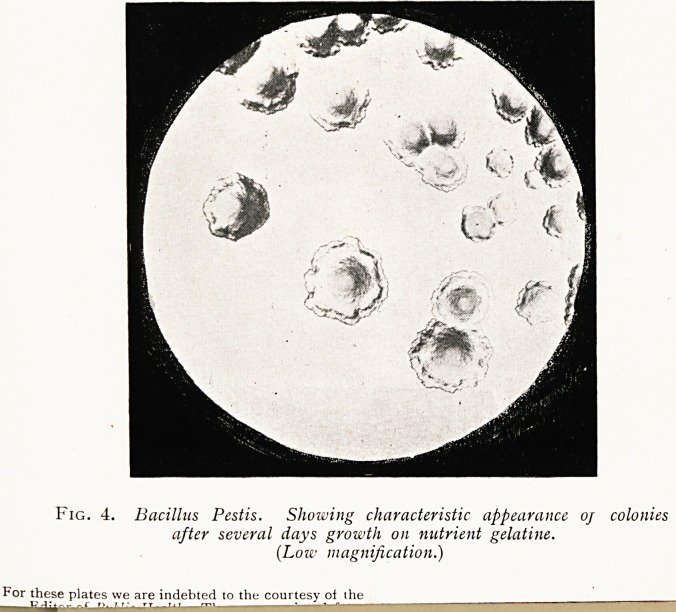# The Diagnosis of Oriental or Bubonic Plague
^1^An address read before the Bristol Medico-Chirurgical Society, March 12th, 1902.


**Published:** 1902-06

**Authors:** E. Klein


### THE DIAGNOSIS OF ORIENTAL OR BUBONIC PLAGUE.[1]

BY

E. Klein, M.D., F.R.S.

---

Within recent years it has become more and more evident that the diagnosis of plague in sporadic cases, from the clinical and pathological appearances, is of a very uncertain nature. This uncertainty is not removed if the case or cases occur in a locality which is in communication by sea or land with a country in which plague is prevalent. Those whose province it is to discover the first cases, in order to institute the necessary measures of precaution against the spread of the disease, are well aware that a febrile rise of temperature, together with acute enlargement of one or the other set of lymph glands, occurring in a person who directly or indirectly has been in some relation to an infected locality, are not sufficient evidence to make the diagnosis of bubonic or Oriental plague.

I am, of course, assuming that other causes—*e.g.* venereal, Hodgkins's disease—are excluded. Real plague is caused by a well-defined species of bacillus—bacillus pestis, which I shall presently have occasion to illustrate fully in its morphological, cultural, and experimental characters. It is admitted that however similar to plague the clinical and pathological features of a given case are, if it does not yield, on competent bacterioscopic analysis, the bacillus pestis, the case cannot be diagnosed as true plague.

Owing to the existence of plague in India, the Cape, Egypt, Arabia, Asia Minor, South America, and other places, it is, of course, a matter of possibility that at any time a ship may bring the infection from those countries into England, and, as is known, individual cases of plague have been so imported, but fortunately have been identified and successfully dealt with, without causing any spread of the infection.

[1] An address read before the Bristol Medico-Chirurgical Society, March 12th, 1902.

With the exception of a single case in Cardiff and a few cases in Liverpool, England and Wales have hitherto not had any internal or endogenous case of bubonic plague. This, I think, affords a striking testimony to the vigilance and circumspection of the Local Government Board, and particularly of the port sanitary and health officers in the different parts of the country.

In this connection, I may be allowed to offer my particular congratulations to the success of the Health Officer of this city, which, by its enormous sea trade, is one of the great portals in which plague might in the natural course of things find an entrance.

Several cases have been notified from different ports which, from their clinical and anatomical symptoms, were suspected to be plague, and which on bacterioscopic analysis were shown to be real plague. Other cases, however, which by their clinical symptoms aroused suspicion, were, on bacterioscopic analysis, shown not to be true plague. I will, for illustration, mention a few of these:—

(1) T. B., St. Bartholomew's. Fever and painful swelling of inguinal glands, particularly on right side; no leukæmia; was bacteriologically proved not to be plague; epidemiologically, mother was in the habit of buying rags in Islington market and bringing them home.

Of the same kind was G. M., Hammersmith. Fever and swelling of glands in neck and axilla and groin; he was a waterman, working a barge carrying timber brought from over sea to London docks. This was a case of streptococcus infection.

In both these cases there was epidemiologically a justifiable suspicion; clinically, the case appeared with symptoms which agreed with those of the bubonic type of plague. Both recovered.

Now, in connection with these and similar cases, it is necessary to consider a theory which has been put forward lately to this effect: In these and similar cases it is said the swelling of the lymph glands, together with a febrile rise of temperature, not being accounted for either by leukæmia,

Hodgkins's disease, or by venereal trouble, represent a disease, not true plague—*i.e.* is not due to the bacillus pestis, but is found in some way or other to occur antecedent to an outbreak of true plague, and for this reason the disease is to be called "pestis minor." I take this opportunity of protesting as emphatically as is in my power against this theory, and to add that, apart from its not being justified, it is mischievous and misleading. If there is one thing well established about plague, it is the fact that it is a specific communicable disease, and is due to a specific well-characterised species of bacillus—the bacillus pestis. To say of a disease, "it is like plague, and it foreshadows plague, but is not itself the true plague, and it is not due to the bacillus pestis, but nevertheless we will call it pestis minor," is, to my mind, meaningless. If the disease is not plague, and is not due to the bacillus pestis—that is to say, if it is some other and different disease,—why call it "pestis" or plague at all?

I may further point out that the Plague Commission sent out to India, to investigate plague, in their report use the term "pestis minor" in the way it ought to be used; viz., to designate the mild form of true bubonic plague, also spoken of as pestis ambulans, in contradistinction to "pestis major" or the severe form of bubonic plague.

(2) A form of plague characterised by severe, acute pneumonia, but not associated with buboes, is from a clinical point of view indistinguishable from other severe forms of pneumonia. Its identification as plague can only be made by bacterioscopic tests. Such cases are of the gravest importance on account of the expectoration and the tissue of the inflamed lung in general containing an abundance of the plague bacilli, and for this reason these cases possess a high degree of infectivity.

A case of acute, severe pneumonia and death within forty-eight hours, occurred in London; it was notified as plague, but bacterioscopic analysis proved it to have been severe typical croupous pneumonia.

(3) A third form of plague is the septicæmic, which cannot be distinguished from a hemorrhagic septicæmia except by bacterioscopic analysis. An instructive case was the following: A nurse in London; she was taken ill on a Thursday, and was dead on the Monday following; owing to an extensive rash and hemorrhage, the attending physician, having seen similar appearances in the East in the septicæmic cases of plague, suspected the case to be one of septicæmic plague. But the subsequent bacterioscopic analysis proved it to have been a case of septicæmia due to the presence abundantly in the circulation of two different microbes, one of which was the diplococcus capsulatus (pneumoniæ), the other a microbe having some points of resemblance to that of bacillus pestis, but easily distinguished from it by culture.

I could multiply these instances to show that it is quite impossible, from a clinical point of view, to diagnose a sporadic case of plague, even when the epidemiological evidence—viz., as to having been directly or indirectly in relation to an infected locality—might point to plague. Not a few times have I had to examine material from persons, who either coming from an infected locality or in connection with a port, showed symptoms of a mild form of bubonic plague, but who, on bacterioscopic evidence and by subsequent events, were proved not to have been so affected. And likewise I have had to deal with materials derived from persons who on clinical grounds were not considered as plague, but on bacterioscopic analysis were proved to have a mild form of bubonic plague, *i.e.* pestis ambulans.

Now, what are the bacteriological facts which characterise true plague, and which enable us in a given case to make the diagnosis of true plague? It must be obvious that the bacteriological tests, in order to be of decisive nature, must be such as not to admit of any doubt or cavil; that is to say, the identification must in all respects be definite—as definite as, for instance, in malignant anthrax or in tubercle.

Now, there are cases in which the diagnosis of plague can without further difficulty be made from microscopic appearances combined with culture:—

(*a*) In the severe form of bubonic plague, very striking and characteristic are the microscopic appearances presented by the swollen lymph glands and the surrounding hemorrhagic tissue of the bubo; a film specimen of the sanguineous exudation is seen to be crowded with short bacilli, as shown in the accompanying lantern slides. The same appearances are presented by the tissue and exudation of the bubo at and near the seat of inoculation in a rodent a day or two after injection of matter derived from the bubo of a case of real plague, as also in the case of rats which have naturally contracted the disease.

In the case of pestis minor (ambulating or sub-acute form), the disease is indicated by the swollen and suppurating lymph-glands; the presence of the bacillus pestis in the exudation and pus cannot easily and definitely be ascertained by microscopic specimens alone, since they are comparatively few and generally amongst crowds of other microbes; here the culture test is indispensable.

(*b*) In acute plague pneumonia (see illustration) the congested lung and the copious sanguineous exudation in it is crowded with the plague bacilli; in cases like those illustrated here (Hull cases) the microscopic examination alone is sufficient to make the diagnosis.

(*c*) In the septicæmic cases of man, and in the fatal natural disease in rats which generally is of the septicæmic type, the blood and particularly the spleen contain the plague bacilli in great numbers; but the bacilli are not to be found in the blood of cases of the bubonic or pneumonic type of man, until the fatal issue.

Calmette's statement that the blood in cases of bubonic plague as a rule contains the plague bacilli is certainly not in accordance with the facts or with the experience of other observers.

*Staining Character of Plague Bacilli.*—Bipolar staining is not characteristic for two reasons: the polar staining is not constant, particularly in culture specimens; nor is it common in the bacilli from the buboes or from the spleen. It seems to me dependent, besides, on certain phases in the life of the microbe. I am inclined to think that the condition of marked polar staining—*i.e.* a vacuole in the centre, the chromatic substance at the poles—indicates rather the reverse of the

FIG. 1. *Bacillus Pestis.* *Stained specimen from juice of acutely inflamed gland.* (× 1000.)

FIG. 2. *Bacillus Pestis.* *Stained specimen from blood of infected animal.* (× 1000.)

FIG. 3. *Pneumonic Plague. Masses of plague bacilli in inflamed lung.* (× 1000.)

FIG. 4. *Bacillus Pestis. Showing characteristic appearance of colonies after several days growth on nutrient gelatine.* (*Low magnification.*)

For these plates we are indebted to the courtesy of the
[illegible]

active vigorous state; whereas the absence of polar staining in the bacilli seems to me to indicate a vigorous and normal protoplasm, uniformly filling the bacilli.

This condition of polar staining is, however, not peculiar to the plague bacillus, since many other bacillary species show this, *e.g.* some species of the colon group, the influenza bacillus, and a peculiar, highly virulent bacillus which I have recently isolated from the blood of a case dead of hemorrhagic smallpox. It would be, therefore, a great risk for any bacteriologist to make a diagnosis of plague merely from the microscopic examination of stained films of a case, say of pneumonia. As a matter of fact, I can quote, besides the cases already quoted, another one of acute pneumonia which was mistaken for plague pneumonia. In this latter the diagnosis of plague was made in a case of acute pneumonia; the diagnosis was based on the presence of numerous polar stained bacilli in the sputum, and from the further fact that guinea pigs subcutaneously injected with the sputum succumbed with hemorrhagic infiltration at the seat of inoculation and enlarged spleen; in the local exudation, as also in the spleen tissue and the blood of the animal, the polar stained bacilli were present in great numbers. But this diagnosis of plague was altogether wrong, because, as was conclusively proved, the bacilli were not those of plague, but a motile, very virulent variety of bacillus coli. I need hardly point out the important financial consequences which would arise if, in any large mercantile centre, suddenly a case of endogenous plague pneumonia were actually notified. It is therefore necessary, before pronouncing on a case, to make quite sure that the diagnosis is based on safe ground.

A second instance of pneumonia suspected to be plague on account of its high infectivity to others living in the same family, was a severe form of influenza pneumonia, the expectoration being crowded with bipolar influenza bacilli; culture and animal experiment left no doubt in the matter.

Numerous lantern slides were shown to illustrate the characters of the bacillus in cultures on various media, gelatine, agar, broth, in plate and in tubes; and the process of agglutination was described.

*Experiments on Animals.*—The plague bacillus and any matter containing it causes on cutaneous or subcutaneous inoculation into rodents a definite acute septicæmic disease, with fatal issue in two, three, or more days. The animals show swelling and hemorrhagic infiltration of the lymph glands and the surrounding connective tissue at or about the seat of inoculation, *i.e.* typical bubo; the exudation of the bubo is crowded with plague bacilli; the blood contains the bacilli, sometimes in relatively small numbers, sometimes in great crowds; the spleen, as also other internal lymph glands are deeply congested and crowded with the plague bacilli. Amongst the animals most susceptible to the disease by subcutaneous inoculation are rodents, guinea-pigs, mice and rats; the rabbit is less easily infected. On account of the more accelerated action the intraperitoneal injection of suspected plague material and plague culture is of greater help in forming the diagnosis rapidly, for after injection of small to moderate doses the animal is dead within twenty-four to thirty hours. On *post mortem* examination the abdominal viscera are found intensely congested, the peritoneal cavity contains viscid grey exudation, which under the microscope is found densely crowded with the plague bacilli, many of them arranged in chains. The intraperitoneal action of b. myxoides was here described. Small grey necrotic patches in the lung, spleen or liver are found in experimental guinea-pigs after inoculation if the disease takes a sub-acute character, *i.e.* death not taking place till about a week or more after inoculation.

It must, however, be borne in mind that although the plague bacillus acts more promptly and in smaller doses in the peritoneal cavity than when injected subcutaneously, the degree of virulence—*i.e.* the amount necessary to cause a fatal issue—is subject to great variations. I possess, for instance, plague cultures which proved of comparatively very low degree of virulence, while others were extremely virulent; thus Oporto and Yeddah cultures, Hull pneumonia, Cardiff rat, London case of 1896, represented various degrees of virulence, the last-named yielding the most virulent cultures.

A point of great interest is the distribution of the bacilli in man and animals affected with plague: in the bubonic type of plague the bacilli are, as a rule, not present in the circulation in the acute stage till shortly before death. In pestis ambulans the suppurating bubo contains few plague bacilli amongst crowds of other microbes, but on account of the discharge of the open sore the danger of spreading the contagion must be considered as real; whereas in the acute stage of the bubonic type this danger—viz., discharge of plague bacilli—is slight or does not exist. In the pneumonic type, on the other hand, owing to the presence of the plague bacilli in the sputum, the danger of infection through the air is a prominent feature. Similarly, owing to the discharge of plague bacilli in all excretions (lung, kidney, alimentary canal) in the septicæmic or hemorrhagic type, the danger of spread of the contagion is very great indeed.

As to animals which have contracted the disease naturally—I refer, of course, principally to rats—the danger of spread of the contagium through these must be considered a reality, on account of the fact that in the rat the natural disease is generally of the septicæmic type; hence plague bacilli can be demonstrated in the excretions, lung, intestine, and kidney. While admitting this, it must not be supposed by any means that this is the principal or the only manner in which plague is spread to and amongst human beings. This is not only not proved, but the existing evidence is directly opposed to it. Professor Koch, in his address to the Congress on Tuberculosis, July, 1901, implies that the whole problem of the importation of plague is of great simplicity; viz., that the rat is the sole instrument by which plague is imported and spread. I consider this something of an exaggeration, and at the same time a statement which is apt to lead to one-sided precautions and faulty steps; for if this view were accepted it would detract the attention of health officers from another real source of danger—real because based on well-observed facts—viz., importation by infected persons and infected articles (clothes, merchandise, &c.).

The Plague Commission in India, while admitting that in some localities outbreaks of plague amongst human beings had been due to rats and in other localities were spread by them, state that in the majority of outbreaks such was not the case, but was due to importation of human cases, or, oftener still, of clothes and effects of persons affected with plague or coming from infected localities. In these instances a mortality of rats from plague was the *result*, but not the *cause*, of plague having broken out in the locality. The first outbreak of plague in Glasgow in 1900 is a good illustration of absence of any evidence as to the rat.

Cardiff is an instructive instance, because it shows (*a*) that a case of plague in man did arise owing to plague amongst rats, and (*b*) that the transmission of plague from the rat to the human being, even if rats die abundantly from plague, is not very high.

While, then, the danger of importation and spread of plague by the rat is a real one, it must not detract our attention from the greater danger of importation and spread of the disease by human intercourse, by clothes and personal effects coming from an infected person or an infected locality.

*Prophylactic Serum and Therapeutic Serum.*—The Plague Commissioners have collected a large body of evidence, both in regard to observations made by competent medical officers in various parts of India, as also in regard to direct observations under their own direction, showing that none of the sera at present in use—Yersin's serum, Lustig's serum—have any therapeutic effect either as to their diminishing the incidence of disease when used prophylactically or reducing the case mortality when used as a therapeutic agent; in fact, the Commissioners rather think the reverse from recommending its use. It has to be remembered that in India in various parts the sera (both Yersin's and Lustig's) were used on a large scale and in various outbreaks, and the Commissioners' conclusions are therefore not merely based on the observations of a few cases in a limited and mild epidemic. This is important to mention, since, for instance, the favourable results which Calmette records in the Oporto outbreak with Yersin's serum, and similar favourable results recorded by Lustig and his assistants in India, are the result of a comparatively limited number of observations gathered not in outbreaks of severe type, but more or less on selected cases of a comparatively mild type.

It is different with regard to injection of Haffkine's plague prophylactic, *i.e.* sterilised plague broth cultures; for as to this the Commissioners have been able in certain localised outbreaks (jails, hospitals) to record definite and positive results, confirming Haffkine's conclusions as to the injection of the prophylactic acting as a real preventive. It both decreases in a marked degree the incidence of the disease, and, failing this, it diminishes the case mortality. The Commissioners add to this that, granted the proper virulence of the plague bacilli with which the broth culture was started, and granted the normal and typical amount of growth in the culture, a single injection of the proper dose of the previously sterilised culture is sufficient. A repetition of the injection, as at first projected, is not required to insure protection.

While all this is admitted, there is another not less important fact which should not be lost sight of—I mean "prevention."

In this the Medical Officers of Health and the Port Sanitary Officers are our best and safest reliance, and it is to them that the real work, and naturally the greatest credit, will have to be assigned. Let us hope that such work and such credit is and will be duly recognised and appreciated.

In the discussion which followed,

Dr. D. S. Davies said: We are all very highly indebted to Dr. Klein for his interesting and practical paper on the bacteriology of plague, and his remarks should teach us to be alert in order to detect an atypical or stray case. But on one point, if I may venture to criticise so high an authority, I must clearly differ from Dr. Klein. He states that in his opinion Koch attached too great importance to the influence of rat infection, and he considers that this induces the danger that Port Medical Officers will neglect due precautions against the other admitted danger of human introduction. I am glad, however, that Dr. Klein definitely admits the reality of rat infection, even though he assigns it a position of subsidiary importance. But, in relying upon the results of the Indian

Plague Committee, who not only made their observations at a date which now renders their report almost ancient history, and at a time when the universal infection of India tended to obscure the lines of causation, he practically ignores the immense additions to knowledge of the past two years, gained in the plague records of newly-attacked countries, and especially in the systematic and convincing report of the Sydney outbreak by Dr. Ashburton Thompson, wherein the slight infectivity of bubonic plague from man to man was clearly demonstrated, and the effective and persistent nature of rat infection was lucidly and forcibly exhibited. Nor has Dr. Klein produced one grain of evidence against the statement of Koch that "in the majority of cases in which the plague has been transmitted by ocean traffic the transmission has taken place by means of plague among the ship rats," or against the view adopted by the Imperial Health Office at Berlin that "plague follows for the most part the routes of the great sea traffic and takes root in seaports, and when introduced by human beings, even under unfavourable hygienic conditions, frequently remains without further consequences, and in those ports where it has developed into an epidemic could hardly ever be traced to the arrival of a plague patient." In other words, he has not disproved the evidence that plague, when it is introduced by persons, may readily be exterminated; when it is introduced by rats, followed by infection of the shore rats, *it has come to stay*. Grain boats appear to be the most dangerous.

The remarkable unanimity with which country after country, when they have had actual experience of plague, adopt stringent and searching measures against rat infection, as evidenced in Australia, in Russia, in Turkey, in Japan, shows how compelling is the prick of experience, and suggests the folly of waiting for similar experience before safeguarding against this admitted and urgent risk. The few English experiences furnish cases in point.

The Hull introductions of 1900 were shipborne, but were all human, and though not at first recognised, and pneumonic, and therefore highly infectious, were yet readily controlled, because the rats were not affected. The Liverpool introductions of 1901 were human, were not for a time discovered, but, as no rat infection had apparently supervened, were readily controlled. The Llandaff case was human; yet, though he travelled across England, he infected no one and led to no outbreak. The Glasgow 1900 outbreak was *apparently* human, and yet, though weeks intervened with some spread, the human outbreak was readily controlled when detected. It is true that some rats then examined did not show evidence of plague, but it was never shown that rat infection might not exist, and the want of a Port Sanitary Authority for Glasgow rendered supervision over ship rats ineffective. Also, the second outbreak of 1901 after some months is quite consistent with the known habits of rat plague: slow, steady spread until the chance of intimate human and rat contact allowed of the outburst of a second human infection, which is in reality a mere fringe of the slow-spreading underground rat epizöotic. The opportunity occurred in the basement of a big hotel, the food department whither rats were attracted, and where they came into intimate human contact, and the usual human outbreak resulted. Examination then showed serious implication existing beyond the hotel precincts, and pointing pretty definitely to a continuous rat infection from the previous outbreak. Plague, having reached the rats, had come to stay: who shall say for how long? There does seem a necessity for intimate association in dwellings in order to secure infection of man from rat. This did not happen in Cardiff as it did in Glasgow, and therefore no human infection resulted.

I do not think there is any risk of Port Medical Officers neglecting human introductions of plague—the existing regulations are clear, stringent, and binding; obscure cases must of course be carefully watched for; but I do think there is risk of rendering the position of Port Medical Officers very difficult, if the misunderstanding should go forth that so high an authority, a trusted adviser of the English Government minimises the importance of this established form of danger. It would render yet more difficult the already most ungrateful task of attempting to exclude plague by keeping infected ship rats from coming on shore, while ships are permitted to carry droves of rats from port to port. We have been through the experience once, and have saved the town from severe pecuniary loss, and the possibility of endemic plague, but we desire no further such experience.

I feel, however, that Dr. Klein's position is not in reality very different from my own; he equally recognises the reality and urgency of rat infection, but he differs as to the relative academical importance of rat and human infection. But Dr. Klein cannot escape from this position:—

If he grants that there is merely a possibility of rats on board ship conveying plague to distant countries, then it must be conceded that their destruction is advisable. But if the belief be not merely that such a mode of extension is possible, but that it is, in all probability, the only dangerous mode of extension, then the destruction of rats becomes a matter of the most vital importance; and the gravest responsibility rests upon Governments and their advisers in opposing any measures which may tend to protect their country against the visitation of this dreaded disease.

The common-sense precaution is to insist, as Australia insists, upon destroying rats *ab initio* in the vessels before loading; this is easily done by fumigation, and periodical attention will keep them free, while precautionary measures are of course taken while loading or unloading at intermediate ports. This policy of rat-free merchant ships has been endorsed by the Turkish Government, and should be internationally agreed to by the European Governments in their own best health interests.

Dr. R. Farrar, who formerly served on the Plague and Famine duty for the Government of India, insisted on the importance of regarding plague infection as primarily an infection of locality. The rat must be regarded as the principal agent in the spread of infection, which is acquired by contact with soil contaminated with the excreta of plague-rats, infection by means of human intercourse being of relatively minor importance. In proof of this contention the speaker pointed out that plague is endemic in certain places, notably at Kumaon at the foot of the Himalayas, and at Shantung in China, etc. When it becomes epidemic the infection is almost invariably seaborne, appearing first in seaport towns. Human epidemics are preceded by the occurrence of epidemic mortality among rats, which are found to be infected with the plague bacillus. Epidemics tend to remain strictly local, instead of spreading rapidly along lines of human intercourse. Thus, Cairo has been known to remain unaffected while plague was raging in Alexandria, and *vice versa*; and of two villages less than 500 yards apart, one might be decimated by plague, the other being exempt, etc. Plague spreads in a town from house to house "like oil on paper," taking weeks or months to spread from one spot to another.

In contending with plague epidemics the policy adopted in India from time immemorial has been that of "evacuation." If infected quarters are evacuated, epidemics are promptly checked, showing that plague is an infection of soil and not conveyed to any great extent by human intercourse. The method of evacuation has been found to be most successful in dealing with the recent epidemic in India. Evacuation checks a crescent epidemic, lessens mortality, shortens the duration of epidemics, and if promptly carried out in villages tends to arrest disease *in toto*. As carried out in India, this policy involves three lines of defence: (1) Hospitals; (2) Observation camps for "contacts"; (3) Health camps. The effect of health camping in Poona was found to be "almost magical." Among six thousand people taken from the worst infected area, only one undoubted case of plague occurred after they had returned to their homes. Immediate disinfection of houses, combined with evacuation, may have a positively harmful effect, from the fact that plague-smitten rats, which would die under the floor of the infected house if left undisturbed, are evicted, and migrate, carrying the infection to neighbouring premises. Disinfection of evacuated houses should be deferred for at least ten days. When isolated cases of plague occur after wholesale evacuation they are nearly always to be traced to the fact of persons secretly visiting their houses against orders, often for the purpose of recovering money which has been buried under the floor. When the soil is infected, plague spreads far more rapidly among bare-footed natives clad in their cotton garments than it is ever likely to do among well-shod and substantially-clad Europeans. Hospital infection is so rare as to be almost a negligeable factor. When cases do occur they are of the pneumonic type.

The inference drawn by the speaker from those and other facts was that plague is always in the first instance a soil infection, conveyed by rats. To guard against this infection of new countries, it is of the first importance to prevent the landing of rats, measures directed to this end being more urgently required than those aimed at the detection and isolation of human cases. Dr. Farrar cordially endorsed the policy of rat-free ships advocated by Dr. Davies.

Dr. SYMES was of opinion that the evidence brought forward in favour of the transmission of the disease from rats to human beings was very untrustworthy and the arguments would apply with equal force to many other diseases, such as typhus fever. He enquired into the resistant powers of the B. pestis and the best methods of staining.

After the discussion, Prof. KLEIN said further :

We are all agreed on the danger of rats, but we are not quite agreed on the point of the relative degree of danger from human beings and from rats; but I feel that rats ought to be destroyed on board all ships.

## Figures and Tables

**Fig. 1. f1:**
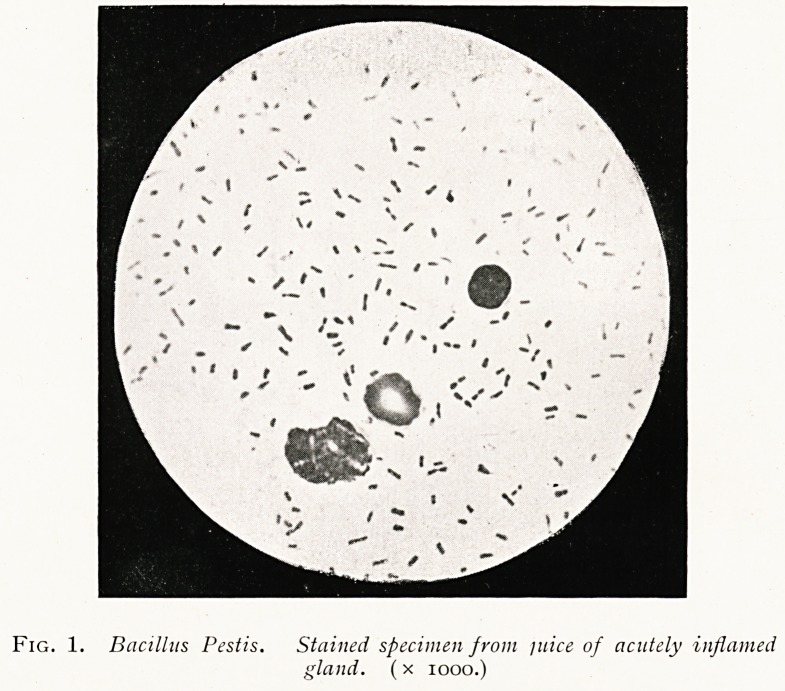


**Fig. 2. f2:**
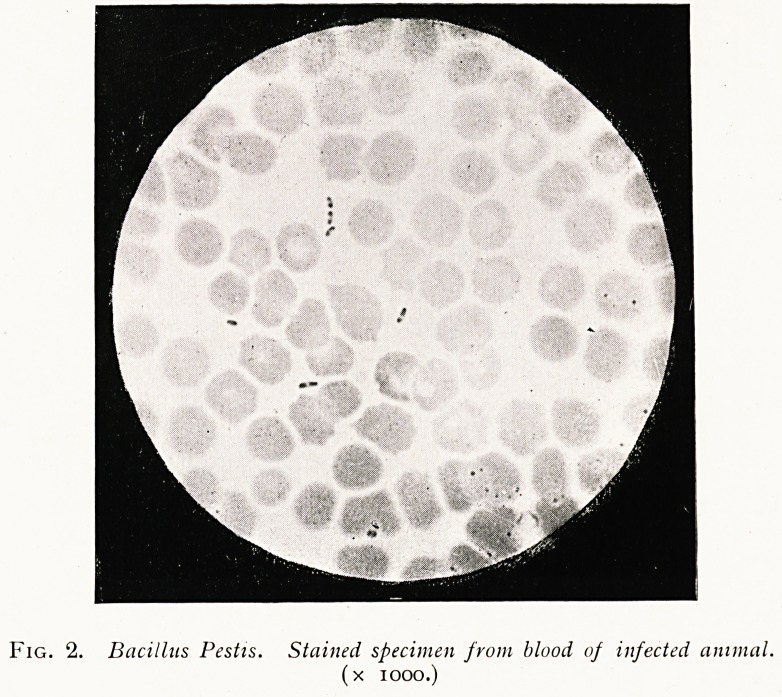


**Fig. 3. f3:**
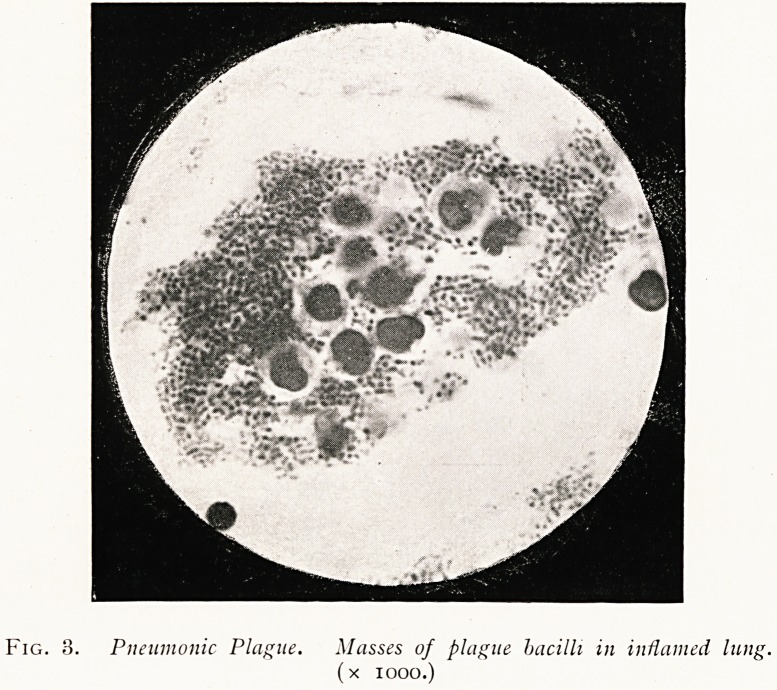


**Fig. 4. f4:**